# Analysis of the lipid extraction performance in a cascade process for *Scenedesmus almeriensis* biorefinery

**DOI:** 10.1186/s13068-020-01870-1

**Published:** 2021-01-14

**Authors:** I. Papachristou, S. Akaberi, A. Silve, E. Navarro-López, R. Wüstner, K. Leber, N. Nazarova, G. Müller, W. Frey

**Affiliations:** 1 Institute for Pulsed Power and Microwave Technology (IHM), Karlsruhe Institute of Technology (KIT), Hermann-von-Helmholtz-Platz 1, Bldg 630, 76344 Eggenstein-Leopoldshafen, Germany; 2 Department of Chemical Engineering, University of Almería, 04120 Almería, Spain

**Keywords:** Microalgae, Lipid extraction, Enzymatic hydrolysis, Pulsed electric fields, Cascade processing

## Abstract

**Background:**

Microalgae have attracted considerable interest due to their ability to produce a wide range of valuable compounds. Pulsed Electric Fields (PEF) has been demonstrated to effectively disrupt the microalgae cells and facilitate intracellular extraction. To increase the commercial viability of microalgae, the entire biomass should be exploited with different products extracted and valorized according to the biorefinery scheme. However, demonstrations of multiple component extraction in series are very limited in literature. This study aimed to develop an effective lipid extraction protocol from wet *Scenedesmus almeriensis* after PEF-treatment with 1.5 MJ·kg_DW_^−1^. A cascade process, i.e., the valorization of several products in row, was tested with firstly the collection of the released carbohydrates in the water fraction, then protein enzymatic hydrolysis and finally lipid extraction. Biomass processed with high pressure homogenization (HPH) on parallel, served as benchmark.

**Results:**

Lipid extraction with ethanol:hexane (1:0.41 vol/vol) offered the highest yields from the different protocols tested. PEF-treatment promoted extraction with almost 70% of total lipids extracted against 43% from untreated biomass. An incubation step after PEF-treatment, further improved the yields, up to 83% of total lipids. Increasing the solvent volume by factor 2 offered no improvement. In comparison, extraction with two other systems utilizing only ethanol at room temperature or elevated at 60 °C were ineffective with less than 30% of total lipids extracted.

Regarding cascade extraction, carbohydrate release after PEF was detected albeit in low concentrations. PEF-treated samples displayed slightly better kinetics during the enzymatic protein hydrolysis compared to untreated or HPH-treated biomass. The yields from a subsequent lipid extraction were not affected after PEF but were significantly increased for untreated samples (66% of total lipids), while HPH displayed the lowest yields (~ 49% of total lipids).

**Conclusions:**

PEF-treatment successfully promoted lipid extraction from *S. almeriensis* but only in combination with a polar:neutral co-solvent (ethanol:hexane). After enzymatic protein hydrolysis in cascade processing; however, untreated biomass displayed equal lipid yields due to the disruptive effect of the proteolytic enzymes. Therefore, the positive impact of PEF in this scheme is limited on the improved reaction kinetics exhibited during the enzymatic hydrolysis step.

## Background

Microalgae have traditionally been part of the diet of various cultures across the globe [1]. After the 1970 oil crisis, they were considered as a potential source for biodiesel production due to their high lipid content, which can reach up to 50% of dry weight [2]. Since then, microalgae have captivated research interest due to their flexible outputs. These microbial factories, depending on their cultivation conditions, can accumulate significant amounts of protein or lipids along with other high value compounds, such as carbohydrates, carotenoids etc. [3]. In relatively simple terms, when the microalgae are cultivated in nitrogen-replete conditions, they are producing proteins. However, upon entering into nitrogen-deplete conditions, they switch to lipid production (additional ways to boost lipid production do exist, however [4]).

Microalgae lipids are composed mainly of triglycerides, which are three long chain fatty acids attached to a glycerol backbone, although other types of lipids can be encountered such as glycolipids or phospholipids [5]. They function either as energy storage or membrane structural components [6]. The length of the carbon chain along with the degree of saturation of the fatty acids directly affects their commercial application. Most microalgae produce saturated or monounsaturated fatty acids, which make an excellent source for biodiesel production [5]. However, certain species can generate significant amounts of polyunsaturated fatty acids (PUFAs) such as docosahexaenoic acid (DHA) and eicosapentaenoic acid (EPA) [7], which are poor feedstock for biodiesel [8] but are of high value for human nutrition and animal feed [9].

Proteins are large biomolecules composed of amino acids and are crucial for the metabolism’s proper function. Microalgae are capable of producing all essential amino acids and in significant portions [10]. This, coupled with high biomass productivity rates and proteins with similar quality to conventional plant-derived ones [11], makes microalgae a potential answer to worldwide growing food demands. Moreover, protein-rich microalgae are considered as a nutrient feedstock by another growing sector, that of biofertilizers [12], due to their potential to enhance crop yields and increased sustainability [13].

Production of high value intracellular components is not enough though, since an efficient and economical extraction technique must also be developed for the commercial deployment. Microalgae proteins are typically extracted through physical or biochemical processes accompanied by recovery of the product through a separation method such as centrifugation and ultrafiltration [14]. Often, to improve the digestibility of the final product, the proteins are hydrolyzed chemically or enzymatically to free amino acids [15]. This strategy is especially effective for biofertilizer production, since it improves the plant’s ability to absorb directly the amino acids by avoiding the protein hydrolysis process, which requires energy [16]. Lipids in contrast to proteins are not water soluble. Their extraction, therefore, requires the use of an organic solvent [17]. An extra challenge is added here since microalgae, unlike land plants, are producing both polar and neutral lipids which in turn requires the usage of a mixture of polar and neutral solvents [18] although successful extractions utilizing only polar solvents (alcohols) have been reported [19]. The traditional solvents used for analytics, chloroform and methanol, are unsuitable for beyond laboratory-scale applications due to their toxicity. Conventional solvents evaluated are ethanol, hexane, acetone and isopropanol among others, with the first two already accepted in the food processing industry [20].

Microalgae cells present a distinctive resistance to intracellular extraction, usually attributed to their rigid cell walls [21]. To overcome this barrier, a pre-treatment method is applied. There are various disruption techniques of different nature and approach (physical, mechanical, chemical) currently evaluated by several research groups as attested by various reviews [22–24]. In principle, the pre-treatment method should be energy efficient, applicable in industrial scales and not harmful to the target compounds. Pulsed Electric Fields (PEF) is a non-thermal technology, which guarantees mild and, therefore, non-damaging operating conditions and has been successfully demonstrated to facilitate extraction of various microalgae intracellular components [25–27]. Even if pilot-scale demonstrations of microalgae treatment are scarce, a number of proven PEF-related applications in the food industry support the applicability of this technology [28–30].

During PEF-treatment, repetitive high voltage pulses of short duration are applied to the microalgal biomass located between two electrodes. The resulting increase of the transmembrane potential leads to the reorganization of the cell membrane and its eventual permeabilization or ‘electroporation’ [31].

Despite the high potential of microalgae, their commercial exploitation remains limited and mostly focused on the production of high value commodities, such as cosmetics or food supplements [32]. While currently being the only profitable options, these applications serve a niche market, easily saturated [33]. A single output process is also gradually phased out in favor of a biorefinery approach. This concept, much like the conventional crude oil refinery, aims towards the complete utilization of the biomass through selective and cascade extraction of different components. A typical strategy would involve the disruption of the microalgae cells, preferably on wet basis to minimize drying costs, followed by the extraction of the water soluble- fraction. Once the aqueous phase is removed, the introduction of an appropriate organic solvent initiates the lipid extraction. The spent biomass can then be further exploited either through gasification for production of energy or directly for animal feed.

The majority of already conducted studies, focused on a single product extraction although some work has been reported on cascade processes as well. Imbimbo et al. performed cascade extraction on the red microalgae *Galdieria phlegrea* with French press as pretreatment method [34]. Proteins along phycocyanin were recovered in the supernatant, whereas carotenoids and lipids were afterwards extracted utilizing pressurized liquid extraction and supercritical fluid extraction, respectively. Francavilla et al. performed lipid extraction from freeze-dried *Dunaliella tertiolecta* with chloroform:methanol followed by fast pyrolysis of the residue. They reported that the resulting bio-oil was in need of upgrading for fuel usage but the produced char could have potential as biofertilizer [35]. Lupatini et al. studied the extraction of proteins and carbohydrates after prior defatting *Arthrospira platensis* with soxhlet extraction [36]. Ansari et al. tested different cascade extraction pathways of different components from dried *Scenedesmus obliquus* concluding that the protein-lipid-carbohydrate route resulted in the optimum recovery of each individual fraction [37]. Interestingly, the authors observed product loss after each step. Another cascade process for *S. obliquus*, was developed by Gilbert-López [38]. In this work, biomass was subjected to High Pressure Homogenization (HPH), followed by freeze-drying. Firstly, triglycerides were extracted through supercritical CO_2_, then various carotenoids were removed with gas expanded liquids and finally pressurized liquid extraction with water was performed for proteins and sugars. In a recent study, Zhang et al. performed a two-stage aqueous and organic extraction from *Nannochloropsis oculata* using High Voltage Electrical Discharges (HVED) and Vacuum Drying (VD) [39]. It was reported that HVED combined with pre-washing had a positive impact on the water-soluble extraction. Moreover, the VD of the spent biomass was accelerated and the subsequent organic solvent of lipids and pigments was improved. The same group also compared HVED and HPH in a multi-step extraction from *Phaeodactylum tricornutum* [40]. HPH proved to be more effective in water-soluble extraction. However, HVED allowed for a more selective aqueous extract and for improved subsequent non-aqueous extraction with chloroform/methanol.

Easy separability of the different fractions is important for reduction of contaminations. PEF offers the advantage of no debris generation [41] since the cells retain their original shape after treatment. Therefore, the integration of PEF in a cascade scheme is a prospect worth examining. A few studies regarding the extraction of multiple products from microalgae with PEF (usually, proteins and carbohydrates) do exist [42, 43] although in these cases, the compounds are extracted together and not in a true cascade format. Guo et al. also evaluated the valorization of residual biomass through hydrothermal liquefaction after the extraction of one product (lipids, proteins or amino acids) [44]. Αn example of cascade extraction of different components from microalgae utilizing PEF, was a previous work of our group [45], where carbohydrate and lipid extractions were performed in two different steps after PEF-treatment of wet *Auxenochlorella protothecoides*.

In a recent study from our group, enzymatic hydrolysis of proteins from wet *Scenedesmus almeriensis* (*S. almeriensis*) was performed after PEF-treatment and compared to HPH treatment as a benchmark. In both cases, the disrupted biomass displayed similar hydrolysis yields and kinetics [15]. *S. almeriensis* is a good candidate for commercial applications since it exhibits significant protein content (50–55% of dry weight) with satisfactory growth rates in a broad range of environmental conditions [46] and is already studied in medium scale production [47]. This microalga has been mainly studied for lutein production and extraction [48, 49]. Bauer et al. performed lipid extraction from *S. almeriensis* among other microalgae with liquefied dimethyl ether although the authors utilized freeze-dried biomass in their study.

**Fig. 1:**
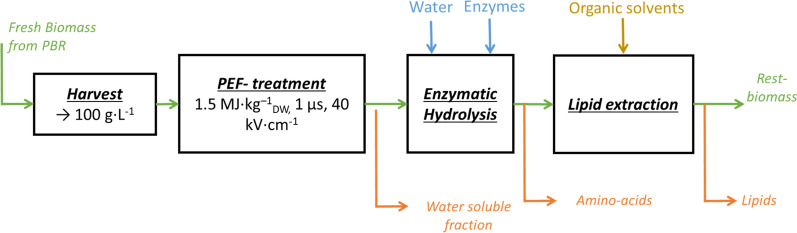
Cascade extraction scheme utilizing Pulsed Electric Fields

A proposed scheme for the serial valorization of several components of *S. almeriensis* is shown in Fig. 1. In this cascade process, the initial step consists of submitting the wet, concentrated biomass to PEF-treatment and evaluating the spontaneous release of intracellular carbohydrates in the water fraction Enzymatic hydrolysis was then performed to cleave and release the intracellular proteins in the form of amino acids in the surrounding aqueous medium. Finally, after removing the water fraction, lipid extraction with organic solvents was performed on the rest biomass.

The goal of this work was to study more specifically the lipid extraction segment of the process described above. Lipid extraction of freshly harvested, wet *S. almeriensis* after PEF-treatment was performed evaluating three different extraction solvents, pure ethanol at room temperature, an ethanol:hexane blend (1:0.41 vol/vol) and pure ethanol at elevated temperature (60 °C). Given the dynamic nature of the PEF effect on cells [45], experiments were conducted immediately after PEF-treatment and after a 24 h incubation step in inert conditions. Once the lipid extraction methodology was established, cascade extraction after 3 h enzymatic hydrolysis was carried out. Thus, a cascade extraction of independent products from wet microalgae utilizing a combination of PEF, enzymatic hydrolysis and lipid extraction could be demonstrated and evaluated.

## Results

### *S*. *almeriensis* composition

All *S. almeriensis* cultivations were conducted in photobioreactors (PBR) for 5 days. The protein, lipid, carbohydrate and ash content of the biomass was determined for each harvest. Total protein content was evaluated through a modified Lowry method (*DC*™, Protein Assay, BioRad) [50], total carbohydrates with sulfuric hydrolysis with Anthrone reagent [45], total lipid content with chloroform:methanol (2:1 vol/vol) in a modified Kochert protocol [51] while inorganics by overnight ashing in a high temperature furnace. Results are presented in Table 1 and indicate that more than half of the biomass, i.e., 55.9% consisted of proteins which made it very suitable for enzymatic hydrolysis.

**Table 1:** Biomass composition of *S. almeriensis*

Composition of biomass	Proteins	Carbohydrate	Lipids	Inorganics	Sum
*S. almeriensis*	55.9 ( ±) 0.53	12.5 ( ±) 2.3	24.2 ( ±) 0.7	5.8 ( ±) 0.14	98.4 ( ±) 3.3

Each component is displayed in percentage of dry weight. Values are the average ± std of three independent cultivations.

### Fatty acid content of *S. almeriensis*

The fatty acid content of the produced biomass was evaluated through gas chromatography after direct transesterification of lyophilized biomass that was priorly molturated with alumine. Results from three independent cultivations are presented in Table 2 and show that *S. almeriensis* is capable of producing noteworthy amounts of polyunsaturated fatty acids.

**Table 2:** Fatty acid content of *S. almeriensis*

Fatty acid	Total fatty acid (%)
14:00	1.8 ± 0.2
16:00	13.8 ± 0.4
16:1n7	4.6 ± 0.5
16:2n4	2.5 ± 02
16:3n4	3.1 ± 0.1
16:4n1	18.2 ± 0.5
18:1n9	6.8 ± 1.8
18:1n7	1.3 ± 0.2
18:2n6	15.8 ± 1.7
18:3n3	30.0 ± 0.7
18:4n3	1.9 ± 0.1
20:5n3	0.1 ± 0.2
Total lipids^a^	23 ± 0.8
Saponifiable lipids^a^	9.1 ± 0.3

Determined with gas chromatography after chloroform:methanol (2:1 vol/vol) extraction of freeze-dried biomass. Results from three independent cultivations

^a^In percentage of biomass dry weight

### Pulsed electric fields treatment of *S. almeriensis*

An indirect but rapid way to determine the efficiency of PEF-treatment on any microalgae suspension is through the conductivity increase measurement due to the release of ions and small charged molecules. After PEF-treatment with 1.5 MJ·kg_DW_^−1^, the conductivity values of the suspension increased by a factor 2, from 1.02 ± 0.1 mS cm^−1^ for untreated biomass, to 2.4 ± 0.1 mS·cm^−1^ for PEF-treated, both values normalized to 20 °C according to Eq. (1). The temperature rise of the suspension due to the Joule effect was equal to 282 K after treatment, with maximum recorded temperature being 33 °C. The conductivity increase after PEF-treatment was in agreement with previous experience performed on this microalgae [15] and confirmed the efficiency of the PEF-treatment.

### Lipid extraction from *S. almeriensis* after PEF-treatment

**Fig. 2:**
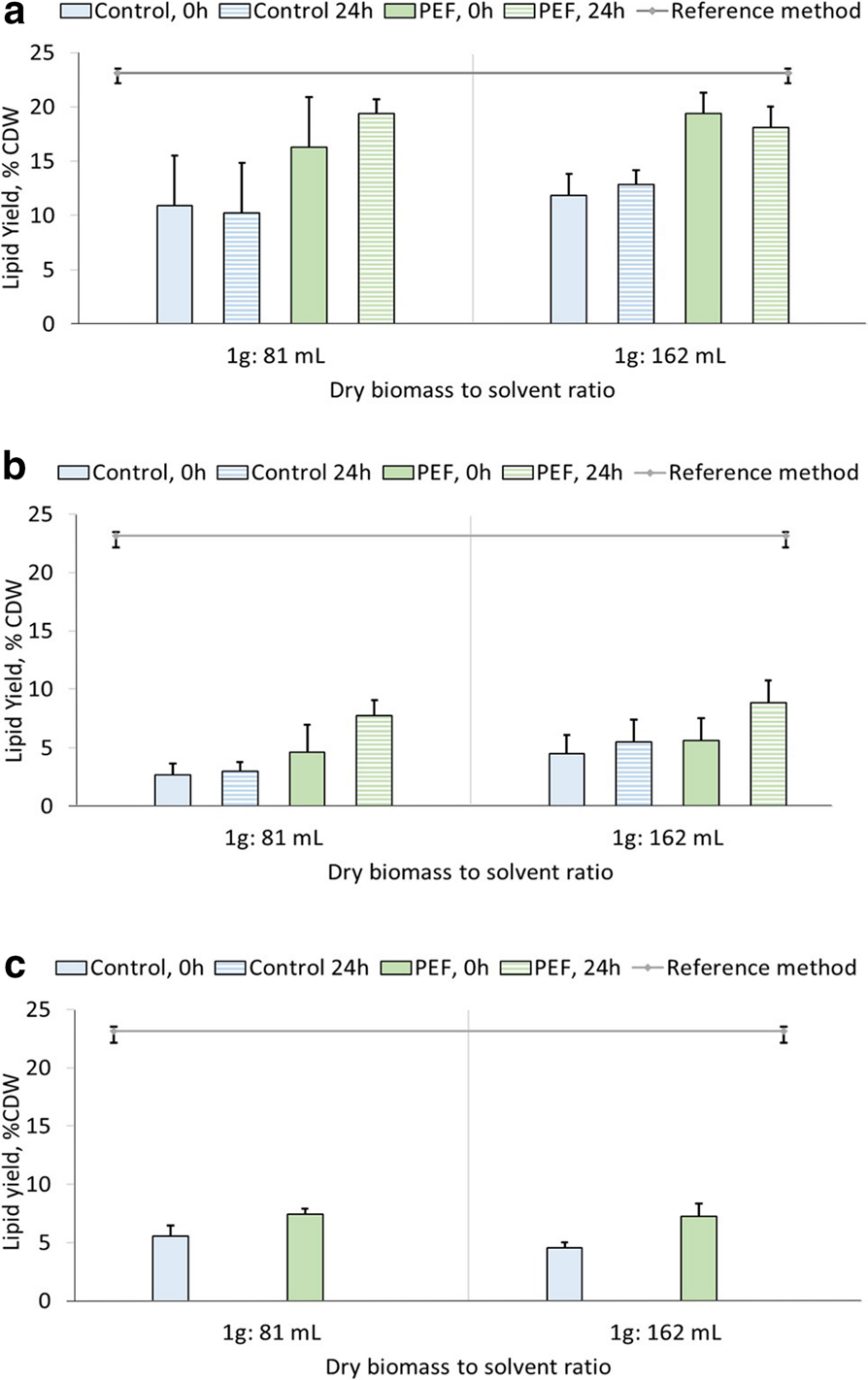
Comparison of lipid extraction from wet *S. almeriensis* after PEF-treatment using three different extraction systems, 24 h extraction with ethanol:hexane at ratios 1:0.41 vol/vol (**a**), 24 h extraction with pure ethanol (**b**) and 0.5 h extraction with ethanol at 60 °C (**c**). In the same graph, the effect of increasing the solvent volume by a factor 2 along with the effect of incubating the biomass for 24 h after PEF-treatment, are shown for systems A and B. In blocks, the average values of lipid yields from two independent experiments are presented along with the standard deviation as error bars. The straight grey line indicates the total lipid content as evaluated from extraction with the reference method, chloroform:methanol 2:1 vol/vol

Three different systems were compared for lipid extraction from wet *S. almeriensis*, namely 24 h extraction with ethanol:hexane (1:0.41 vol/vol), 24 h extraction with pure ethanol and 0.5 h extraction with pure ethanol at 60 °C. As mentioned in Sect. 5.4., the protocols were adapted from literature and were proven effective for lipid extraction from other microalgae strains. The effect of solvent volume was additionally examined by increasing it by a factor 2. For the first two extraction systems, experiments were also conducted on biomass that was incubated for 24 h after PEF-treatment. In Fig. 2, the results from two independent cultivations are presented along with standard deviation.

As it can be seen for system A, direct extraction from untreated biomass offered 10% yields on dry weight, which would correspond to 43% of the total lipid content as estimated by the reference method. PEF-treatment with 1.5 MJ·kg_DW_^−1^ followed by immediate lipid extraction offered an increase of the lipid yields, up to 16.3% in dry weight or 70% of the total lipids. PEF-treatment thus increased the yields by almost 60% compared to control biomass. Increasing the solvent amount by a factor 2 did not increase the yields from untreated biomass and offered a slight increase of 3% dry weight for PEF-treatment. Incubating the biomass for 24 h after PEF-treatment, results in a very similar yield increase by 3% dry weight, with Control samples remaining unaffected.

For pure ethanolic extraction (system B), lipid yields were relatively low for all conditions tested. More specifically, untreated biomass never exceeded 5% of lipid yields in dry biomass. PEF-treatment offered similar yields with incubation after PEF-treatment, offering a borderline increase by 3%. Interestingly enough, both solvent volumes offered almost identical results.

System C, ethanolic extraction at 60 °C painted a similar picture with the previously discussed system. Only 7% lipid yields were achieved with PEF-treatment, less than 30% of the overall lipid content. Solvent volume did not affect the yields. For this experiment, no incubated biomass was tested.

Even though yields never reached the ones obtained from freeze-dried and bead milled biomass, the extraction system utilizing ethanol:hexane was selected to be applied after the enzymatic hydrolysis since based on the results from Fig. 2, it offered the highest yields.

### Cascade processing of *S. almeriensis*

The selected lipid extraction protocol was then tested within the context of a biorefinery scheme. Biomass treated with PEF at 1.5 MJ·kg_DW_^−1^ without any incubation was tested along with untreated microalgae. HPH served as benchmark for comparison.

#### Water fraction

**Fig. 3:**
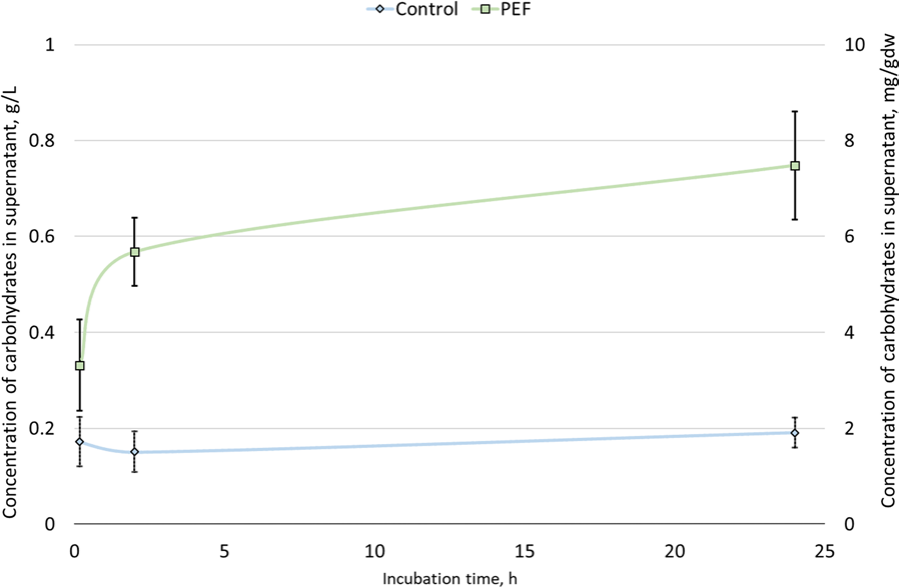
Carbohydrate release from *S. almeriensis* in the supernatant after PEF- treatment, with no incubation, 2 h incubation and 24 h incubation. Results of two independent cultivations are displayed in average with the error bars indicating the standard deviation. On left *y-axis*, results in g carbohydrates per L while on the right *y-axis* mg carbohydrates per g dry weight

The potential valorization of the water fraction was examined by evaluating the intracellular release of carbohydrates after PEF-treatment as observed on a previous study on *A. protothecoides* [45]. The microalgae suspension was centrifuged immediately after, 2 h or 24 h after PEF-treatment. The spontaneous carbohydrate release in the supernatant over these time points was determined with the Anthrone method as descripted in session 5.2.3. The results from two independent cultivations are shown in Fig. 3. As it can be seen, the carbohydrate concentration in the supernatant of untreated samples remained stable at 0.2 g L^−1^. Treatment with PEF offered a slight release immediately to ~ 0.3 g L^−1^ which almost doubled after 2 h incubation and reached up to ~ 0.8 g L^−1^ after 24 h.

#### Enzymatic hydrolysis

**Fig. 4:**
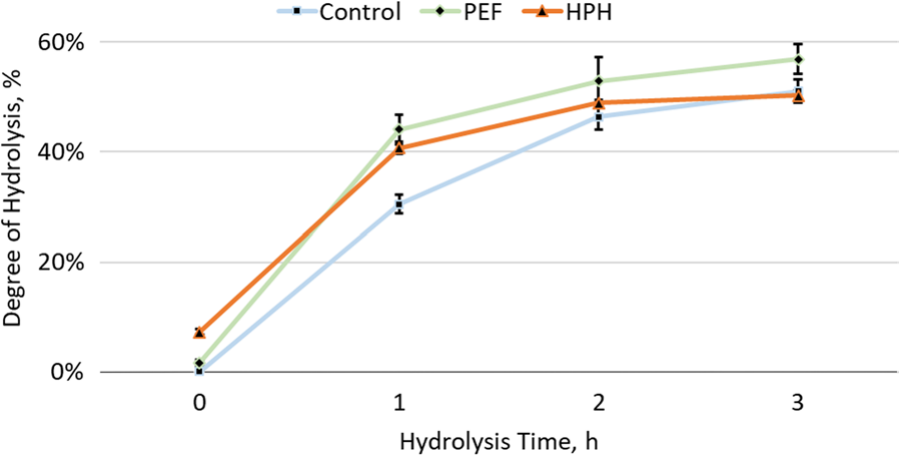
Kinetics of the enzymatic hydrolysis of wet *S. almeriensis.* Biomass was either untreated (Control), fed into high pressure homogenizer (HPH) or treated with pulsed electric fields, using 3% enzymes (vol/w). The average of three independent cultivations are presented with standard deviations as error bars

Enzymatic hydrolysis of wet *S. almeriensis* took place for 3 h at 50 °C, at controlled temperature and pH. As benchmark for comparison to PEF, HPH was also tested. At the end of the reaction, the samples were centrifuged to separate the aqueous phase along with the free amino acids and the degree of hydrolysis was determined. The kinetics of the enzymatic hydrolysis over 3 h are presented in Fig. 4.

At 0 h right before the addition of the enzymes, HPH-treatment released higher amounts of free amino acids with a degree of hydrolysis equal to ~ 7% against PEF-treatment, which released 1.7% into the suspension. It has to be mentioned that in untreated biomass no amino acids have been detected which confirms that the washing step had no effect on the cells. After 1 h of hydrolysis, PEF-treated samples displayed a similar degree of hydrolysis as HPH at 44% and 40%, respectively. Untreated samples were still lagging behind with a degree of hydrolysis of 30%. After 2 h of reaction, the hydrolysis was slowly reaching equilibrium. At this time point, PEF-, HPH- and untreated samples had a degree of hydrolysis of 53%, 49% and 46%, respectively. With a prolongation of the reaction, up to 3 h, the degree of hydrolysis for PEF-treated biomass increased up to 57%, Control reached 51%, whereas HPH samples remained unaffected.

#### Lipid extraction after enzymatic hydrolysis

**Fig. 5:**
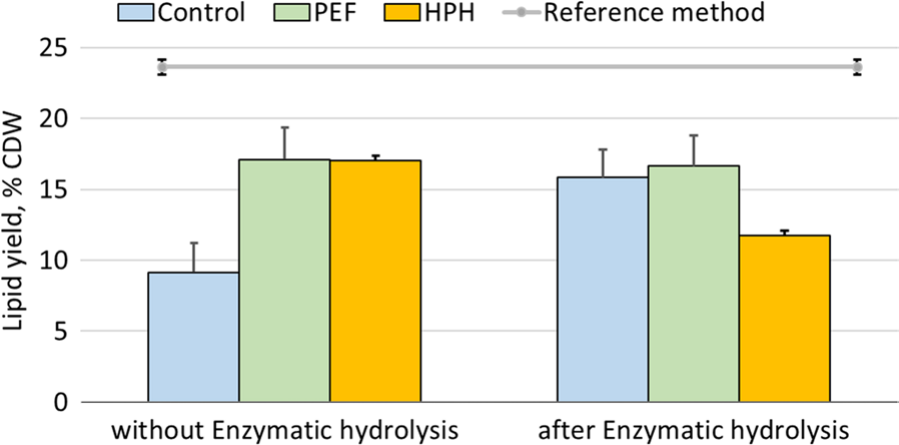
Lipid extraction from wet *S. almeriensis* following enzymatic hydrolysis within a cascade processing. Biomass was either untreated, fed into high pressure homogenizer or PEF-treated. Lipid extraction was performed either directly after pre-treatment or after enzymatic hydrolysis. In blocks, the average values of lipid yields from three independent experiments are presented with standard deviations as error bars. The straight grey line displays the total lipid content as evaluated from chloroform:methanol (2:1 vol/vol)

The residual biomass was then subjected to lipid extraction to evaluate the feasibility of cascade processing. The results from three independent cultivations are shown in Fig. 5.

Lipid extraction from fresh *S. almeriensis* without enzymatic hydrolysis behaved in a similar manner with the results described before in Fig. 2. Untreated biomass had on average 10% lipid yields on dry weight, whereas PEF-treated samples exhibited increased yields, up to 17%. HPH was also highly efficient, displaying similar yields, equal to 17% dry weight. When extraction was performed in cascade after enzymatic hydrolysis, the lipid yields of PEF-treated samples were not affected. However, a sharp increase in the yields of the untreated sample before the enzymatic hydrolysis was observed. Indeed, as shown in Fig. 5, the yields had risen up to ~ 17% of biomass dry weight, i.e., very similar to samples initially pre-treated with PEF. In contrast, if the biomass is treated with HPH before the enzymatic hydrolysis, the lipid yields after extraction of the rest biomass are lower compared to previous conditions, namely a little higher than 10% of dry weight.

## Discussion

The produced biomass was rich in protein content (more than 50% of dry weight), with lipids being the second higher component with approximately 24% of dry weight. Only 12% of carbohydrates were produced, while the non-volatile inorganics made up 5–6%. The overall mass balance was close to 100% to a satisfactory degree (98.4%). The composition was slightly different compared to *S. almeriensis* as reported by Romero García et al. with values 41.8%, 11.2%, 38.7% and 8.3% for proteins, lipids, carbohydrates and ashes, respectively [52]. In that study, however, the biomass was cultivated in continuous mode with daily harvests and in Mann and Myers medium instead of Arnon medium used in the current study, which could explain the differences.

Approximately 40% of the fatty acids of the produced biomass were saponifiable, an important parameter regarding their potential for biodiesel. Moreover, the lipids were rich in polyunsaturated fatty acids, namely alpha-Linolenic acid C18:3n3 (30% of total fatty acids) and linoleic acid C18:2n6 (15.8% of total fatty acids). This might make such lipids unsuitable for biodiesel production since polyunsaturated fatty acids are considered bad feedstock [53, 54], but are highly valued in the nutrient sector. As reported by Ruiz et al., microalgae for food has three times higher potential value compared to other biomass usages [33]. Kumar et al. also state that lipid extraction from even microalgae strains with lipid content as little as 10% will be feasible given a large enough production [55]. Therefore, the composition of *S. almeriensis*, reported in this work with high protein and modest polyunsaturated lipid amounts, was deemed appropriate for the cascade process examined here.

As shown in Fig. 2, PEF-treatment promoted lipid extraction but only in combination with the appropriate solvent. The ethanol:hexane blend (1:0.41 vol/vol) that was successfully applied for lipid extraction from *A. protothecoides* in a previous work [56], offered the highest yields in this study. On the other hand, utilizing only ethanol, the yields were significantly lower, both for untreated and PEF-treated samples. These results are in agreement with a study from Sean Lai et al. who performed lipid extraction using chloroform:methanol, pure ethanol and pure hexane after subjecting *Scenedesmus *spp. in a double pass through PEF with an overnight storage and subsequent freeze-drying. The authors reported that ethanol offered the lowest yields from the systems examined, while the co-solvent approach was favored by PEF-treatment [57]. Pure ethanolic extraction is promoted as a viable alternative for lipid extraction as seen in Yang et al. who achieved high lipid yields from the wet microalga *Picochlorum sp* [19]. Navarro López et al. using only ethanol were able to successfully extract lipids from wet *Nannochloropsis gaditana* [58], whereas in this study using a similar methodology (extraction system C) only 30% of the total lipids were extracted. Yao et al. also reported an effective usage of isopropanol as lipid solvent from *Nannochloropsis sp*. at 80 °C [59]. The same authors state that elevated temperatures favor extractions with alcohol. In this study, a comparison of systems B and C, i.e., ethanol at room temperature versus 60 °C, resulted in slightly increased lipid yields for the latter. Elevated temperatures generally favor the extraction of thermally stable products, which could explain this difference [60] although this might lead to damage of the unsaponifiable fraction. From the above it can be concluded, that PEF can serve as a pre-treatment method from *S. almeriensis,* however, a mixture of polar and neutral solvents, in relatively large and potentially unsustainable volumes, is necessary for effective lipid extraction, in this case ethanol:hexane. Whether the necessity to use a co-solvent is due to the microalgae structure, e.g., the cell wall composition, or whether it is imposed by the lipid type, was not investigated in this study.

Regarding the long-term effect of PEF-treatment, the lipid yield was higher when microalgae were incubated for 24 h before submitted to lipid extraction. Indeed, 83% of total lipids were extracted with 24 h incubation compared to 70% without incubation. A similar tendency was observed in previous experiments with *A. protothecoides* [45]. It is peculiar though that independently of the state of the biomass (fresh or 24 h incubated), 100% total lipid extraction was never achieved compared to the reference method. A significant increase of the solvent volume by a factor 2 had practically no effect on the lipid yields, indicating that the solvent volume was not the limiting factor. The reference lipid extraction was performed on freeze-dried biomass after bead milling which completely shatters the microalgae. As shown in Fig. 5, the lipid yields of HPH-treatment samples were very similar with PEF-treated ones. This fact implies that this inability to reach total lipid extraction was not because of the pre-treatment itself but instead due to the limitation of the solvation ability of ethanol:hexane, potentially further reduced by the presence of water. A second possible explanation would be a slight overestimation of lipid yields with the reference method. In a chloroform:methanol extraction process, once the system is turned biphasic, the lower phase containing the lipids is separated from the upper phase by pipetting. It has to be considered that this pipetting step carries an increased risk of contaminations from the upper phase [61]. In ethanol:hexane systems though, the hexane along the lipids form the upper phase, resulting in a more clean separation without any contaminants in the gravimetric determination.

Regarding the spontaneous release of other compounds into the water fraction after PEF-treatment during the first step of a cascade process, only small amounts of carbohydrates were detected in the supernatant, up to ~ 0.8 g·L^−1^. For comparison, in a previous study with *A. protothecoides* up to 8.0 g·L^−1^ of carbohydrate were released in the supernatant after PEF treatment of a 100 g·L^−1^ suspension [45]. One explanation for this different behavior could be due to the very different composition of the two microalgae. Indeed, *S. Almeriensis* had a relatively low total carbohydrate content, i.e., ~ 12.5% dry biomass, while *A. protothecoides* ranged between 20 and 30% dry biomass (unpublished observations). Another possible scenario could be that *S. Almeriensis* responds less efficiently to the PEF-treatment regarding the release of intracellular soluble molecules. The cell wall of *Scenedesmus* strains contains an additional pectin layer compared to *Chlorella* species [62] so it is possible that this additional barrier hampers any intracellular component diffusion. Nonetheless, the fact that only little amount of carbohydrates were detected, does not render the water fraction necessarily without value. The biostimulant activity of the supernatant after PEF-treatment could be evaluated in a similar study like Navarro-López et al. [63].

Concerning the enzymatic hydrolysis, the results were in agreement with data previously reported from our group with samples that underwent HPH exhibiting slightly lower yields [15]. This discrepancy was attributed to the fact that HPH treatment in the previous work was performed at concentrations of 50–80 g·L^−1,^ whereas in this study it was conducted at 100 g·L^−1^ which resulted in a reduced efficiency using our apparatus. PEF-treated samples though were not affected by this increase of concentration.

The lipid extraction that was performed after enzymatic hydrolysis gave interesting insights on the possibility of combining the two processes in a cascade. From Fig. 5, it can be derived that for PEF-treated samples, the enzymatic hydrolysis has a minimum impact on the lipid content and the lipid yields. However, while PEF-treatment was beneficial for lipid extraction, it appears that at the end of the enzymatic hydrolysis reaction, the untreated biomass displayed similar lipid yields as the PEF-treated one. It is apparent, that enzymatic hydrolysis acted as a pre-treatment with the enzymes damaging the cell wall [64], resulting in an increased subsequent lipid extraction. The HPH-treated lipid yields after enzymatic hydrolysis were less compared to both untreated and PEF-treated samples (11% versus ~ 16% dry weight). The destruction of the cells and the resulting emulsification of the various cell components could have led in lipid losses during the removal of the aqueous phase at the end of the enzymatic hydrolysis something, which might explain this observation. While the above could be interpreted as proof that PEF is applicable in a cascade process, the increased lipid yields displayed by untreated samples after enzymatic hydrolysis, render the effect of PEF-treatment moot as far as the lipid extraction is concerned. Any potential benefits from PEF-treatment in this scheme, therefore, have to be detected in the possible valorization of the water fraction or in the improved kinetics displayed during the enzymatic hydrolysis.

## Conclusions

In this study, three different lipid extraction systems were carried out on wet *S. almeriensis* biomass that was pre-treated with PEF at 1.5 MJ·kg_DW_^−1^. Among the three extraction systems (ethanol:hexane, pure ethanol at room temperature and pure ethanol at 60 °C) that were tested, ethanol:hexane clearly displayed the best performance, with 70% of total lipid content extracted (increased up to 82% of total lipids if a 24 h incubation step is introduced after PEF-treatment) with a clearly positive effect of PEF observed.

The utilization of PEF in a biorefinery processing of *S. almeriensis* composed of a water fraction extraction, an enzymatic hydrolysis and a lipid extraction was examined and compared to HPH as benchmark. Very little amounts of spontaneously released carbohydrates were detected in the water fraction after PEF-treatment. During the enzymatic hydrolysis, PEF and HPH accelerated the reaction kinetics in an equal manner. In the subsequent lipid extraction, however, PEF-treated samples retained their high lipid yields in contrast to HPH-treated biomass, which displayed diminished results. The most likely explanation for this observation is the complete cell fractionation after HPH treatment and the lipid losses in the formed agglomerates. However, completely untreated samples displayed equal lipid yields with PEF after hydrolysis, limiting thus any positive impact of PEF to the eventual valorization of the water fraction or to the improved reaction kinetics exhibited during the enzymatic hydrolysis step.

## Materials and methods

### Cultivation and harvest of biomass

The cultivation conditions of *S. almeriensis* were identical to the description provided in [15]. In brief, the biomass was cultivated in Arnon medium in a 25 L bubble column annular bioreactor illuminated 24 h at 250 μ·mol^−2^·s^−1^ with temperature maintenance at 25 °C. Supply of air and CO_2_ was provided at a rate of 5000 cm^3^·min^−1^ and 25 cm^3^·min^−1^, respectively. The pH was fixed at 8. The cultivation lasted for 5 days.

Harvest was carried out using a separator (STC 3–06-170, GEA Westphalia, Germany). The resulting biomass paste was re-suspended with deionized water with a twofold purpose. First, to adjust the biomass concentration at ~ 100 g·L^−1^, i.e., as high as possible to reduce the energy input of the PEF-treatment. It has to be considered that the biomass needs to be still liquid enough to be pumped through the PEF treatment chamber. Second, to reduce the conductivity of the microalgae suspension from the initial 4.2 mS·cm^−1^ down to 1–1.2 mS·cm^−1^. The obtained conductivity corresponds to the design parameters of the treatment chamber for matched conditions and, therefore, ensures square electric pulses. As shown in the previous study, *S. almeriensis* is resistant to any osmotic shock resulting from this washing step [15]. The exact final concentration was determined by overnight drying of known amounts of the final suspension and supernatant in a drying oven (Universalshrank model U, Memmert, Germany) [40]. After each harvest, part of the biomass would be freeze-dried and stored in vacuum-sealed bags at -20 °C for composition determination of the biomass. Freeze-drying was conducted in a laboratory freeze-drier (Alpha 1–4 LDplus, Christ) for at least 24 h and stored afterwards in vacuum-sealed bags.

### Biomass composition characterization

After each harvest, the composition of *S. almeriensis* was determined and more specifically, the total protein, carbohydrate, lipid and inorganic (ashes) content. Protein determination took place in fresh microalgae, while all the other analyses were performed on freeze-dried biomass.

#### Total protein determination

To determine total protein content, a chemical extraction was performed using sodium hydroxide. From concentrated suspension, a volume that contained 5 mg of microalgae biomass was resuspended in 2 mL sodium hydroxide (1 M), followed by 1 h of incubation at 95 °C. After this step and upon reaching room temperature, the sample was centrifuged at 10,000×*g* for 10 min and the supernatant was processed for protein determination with a modified Lowry method (DC™ Protein Assay, BioRAd) using bovine serum albumin as standard [50].

#### Total lipid determination

Chloroform:methanol extraction was performed on freeze-dried *S. almeriensis* utilizing a modified Kochert protocol to determine the total lipid content [51]. Freeze-dried biomass was bead-milled at 30 Hz, 5 times for 15 s (Mixer mill, MM400, Retsch, Haan, Germany) and approximately 100 mg were recovered and measured in a precision balance. 2 mL of chloroform:methanol (2:1 vol/vol) were mixed with the biomass, vortexed and immediately centrifuged at 1800×*g* for 4 min. After the centrifugation, the supernatant was removed and collected into a separate glass tube. 2 mL of fresh solvent were added in the biomass and the above process was repeated. Overall, 7 mL of solvent were used, in four separate extraction steps (3 × 2 mL and 1 × 1 mL for the last step). In the glass tube with the collected solvent, 3 mL of HCl 0.1 N and 0.3 mL MgCl_2_ 0.5% were added to facilitate phase separation. The lipid-containing lower phase was removed with a Pasteur pipette into pre-weighted glass tubes and evaporated under N_2_. The lipid yield was determined gravimetrically. All samples were performed in duplicates.

#### Total carbohydrate determination

The determination of carbohydrate release was conducted using the Anthrone sulfuric acid assay [45]. Freeze-dried biomass was resuspended in deionized water in concentrations ~ 0.1–0.2 g·L^−1^. On parallel, fresh starch aqueous solutions with concentrations ranging from 0.02 g·L^−1^ to 0.4 g·L^− 1^ were prepared from starch powder (Merck 1.01257) to be used as standards and processed in a similar manner with the samples. The anthrone reagent was prepared on the day of the experiment by dissolving anthrone (Merk 1.01468) in 95% sulfuric acid (AnalaR NORMAPUR: VWR Chemicals 20,700) at a final concentration of 0.1% w/v. 400 μL of diluted sample or standard along 800 μL of anthrone reagent were mixed in 1.5 mL Eppendorf Safe Lock tube. After 5 min incubation in ice, the sample was placed into a thermo-incubator pre-heated at 95 °C and shaken at 300 rpm for 16 min followed by cooling down on ice for again 5 min. Optical density of the cooled samples was measured at 625 nm in a spectrophotometer (Genesys 10S UV–Vis, Thermo Scientific) and carbohydrate concentration was calculated using the standard curve and considering the dilution factors. All measurements were performed in duplicate.

#### Inorganic components measurement (ashes)

Approximately 200 mg of freeze-dried biomass were measured in a precision balance in alumina crucibles and placed in a high temperature furnace (Hochtemperaturofen Supertherm HT04/17, Nabertherm, Germany) for overnight ashing. After removal from the furnace, the samples were let to cool down to room temperature, whereupon they were measured again in the precision balance. The ash content was determined gravimetrically and in duplicate.

### PEF-treatment and incubation of biomass

PEF-treatment of freshly harvested biomass took place in a custom-made continuous flow treatment chamber with a 4 mm distance between the electrodes. The apparatus was identical with previous works. The generator was described in [56] and photos of the chamber and electrodes are available in [65]. In brief, the pulse parameters were set to duration of Δ*t* = 1 μs, electric field intensity of 4 MV·m^−1^ and repetition rate of 3 Hz with a constant flow microalgae rate in the treatment chamber equal to 0.1 mL·s^−1^. These parameters correspond to an energy input of 150 kJ·L^−1^ or 1.5 MJ·kg^−1^_DW,_ treatment, conditions that are demonstrably effective to *S. almeriensis* and to other microalgae based on our previous works [15, 56]. Further details on the estimation of the energy input can be found in [45].

The conductivity value of the microalgae suspension before and after PEF-treatment was measured with a conductivity meter (WTW, cond 3310), without automatic temperature compensation. The conductivity values were normalized to 20 °C using the Eq. (1), where σ stands for the conductivity, T for the measured temperature and α20 the temperature compensation coefficient at 20 °C which is equal to 2.38% per degree of centigrade [15].

1 $${\sigma }_{20}={\sigma }_{T}\frac{1}{1+{\alpha }_{20}(T-20)}$$

### Lipid extraction from wet *S. almeriensis*

The lapsed time after PEF-treatment has been demonstrated to be an important parameter that can directly affect the lipid extraction yields [45]. Therefore, lipid extraction experiments were performed on biomass immediately after PEF-treatment and after an incubation step. During this incubation, both PEF-treated and untreated biomass were stored in inert conditions (flushed with N_2_, in dark, without any agitation) for 24 h prior to further handling.

Three different protocols were tested for lipid extraction from wet *S. almeriensis*. The first one was a co-solvent ethanol: hexane (1:041 vol/vol) system (system A), adapted from *Grima et al. *[66]. The second system was pure ethanol (system B) adapted from Eing et al.[67]. Both these protocols were priorly proven to be very robust for lipid extraction from *A. protothecoides* [56, 67]. The third one, pure ethanol in elevated temperature (system C) was adapted from Navarro-López et al.[58] who demonstrated its effectiveness for lipid extraction from *Nannochloropsis gaditana*. The protocols are summarized in Table 3 and described in detail below. All reagents were of analytical grade. Two independent cultivations were studied, with each sample processed in duplicate.

**Table 3:** Composition of the different extraction systems

Extraction system	Ethanol mL/ 1 g dry biomass	Hexane mL/ 1 g dry biomass	Water mL/ 1 g dry biomass	Duration of extraction (h)
Ethanol:hexane (1:0.41 vol/vol) (System A)	54	22	~ 5	24
Ethanol (System B)	76	-	~ 5	24
Ethanol elevated at 60 °C (System C)	76	-	~ 5	0.5

Values are given in mL, normalized to 1 g of dry biomass. Water is left from the dewatering step and is subject to slight variations

#### Ethanol:hexane extraction (system A)

For each sample, approximately 3 mL of suspension were measured in Teflon tubes (Nalgene® Oak Ridge Centrifuge Tubes, Teflon® FEP, 50 mL Thermo Scientific), which at 100 g·L^−1^ concentration corresponds to 0.3 g biomass. The probes were then centrifuged (Heraeustrade; Megafugetrade 8R, ThermoFischer Scientific, Germany) at 10,000 × *g* for 10 min and the supernatant was removed and measured to evaluate the remaining water in the system which was equal to approx. 1.5 mL. The biomass pellet was then re-suspended by adding 16.1 mL ethanol and 6.6 mL hexane. The composition of the system at the beginning of the extraction was thus 1:0.41:0.09 ethanol:hexane:water with a ratio of 81 mL of solvent per 1 g dry weight. It should be noted, that the water present is the leftover in the biomass pellet from the above centrifugation step, without any extra water addition at this stage.

After rigorous vortexing, the samples were left to agitate on an agitator for 24 h, in the dark and at room temperature. Once extraction was completed, the probes were centrifuged at 10,000 × *g* for 10 min, to separate the solvent from the residual biomass. From the supernatant, 6.1 mL were removed into a separate tube, where an additional 18.2 mL hexane and 2.9 mL distilled water were added. From the two distinct phases formed, 15 mL from the upper, hexane lipid-rich phase was removed into pre-weighted glass tubes and evaporated under N_2_. The lipid yields were then calculated gravimetrically. Incubated samples were treated in an identical manner.

#### Ethanol extraction (system B)

The samples were prepared in a similar manner with the previous protocol up to the addition of the solvent, where instead of the ethanol:hexane blend, 22.7 mL of pure ethanol were added resulting in a ~ 96% ethanol extraction system at a ratio of 81 mL of solvent per 1 g dry biomass. Extraction took place in the dark, on an agitator for 24 h. The extraction solvent was separated by the spent biomass by a 10 min centrifugation at 10,000 × *g*. From the supernatant, 11.4 mL were removed into a separate tube, where 11.4 mL of hexane and 5.7 mL 10% NaCl deionized water were added. From the two phases formed, 9 mL of the upper phase were removed into pre-weighted glass tubes and evaporated under N_2_. The lipid yields were then calculated gravimetrically. Incubated samples were treated in an identical manner.

#### Ethanol extraction at elevated temperature (system C)

Like the previous systems, 3 mL of concentrated microalgae suspension were measured in a precision balance in teflon tubes and further de-watered after centrifugation. The biomass pellet was re-suspended by adding 22.7 mL pure ethanol resulting in a ~ 96% ethanol extraction system at a ratio of 81 mL of solvent per 1 g dry biomass. Extraction took place in the dark, for 30 min at 60 °C in a water bath (SONOREX SUPER RK 510 H, Bandelin, Germany). Following this, the samples were centrifuged at 10,000 × *g* for 10 min. The supernatant was completely removed into a separate tube, where 6.81 mL hexane and 13.6 mL deionized water was added. After the formation of two distinct phases, 5 mL from the upper phase was removed into pre-weighted glass tubes and evaporated under N_2_. The above step was repeated with the addition of 5 mL fresh hexane. Gravimetric yields were measured gravimetrically.

#### Increase of the extraction solvents by factor 2

Experiments were conducted, where the extraction solvent was increased by a factor 2. The protocols followed were identical to the description above, the main difference being that instead of 3 mL sample, 1.5 mL were measured instead, corresponding to 162 mL solvent per 1 g of dry biomass.

#### Lipid transesterification and gas chromatography analysis

To evaluate the saponifiable content of *S. almeriensis*, the biomass underwent a direct transesterification reaction, followed by gas chromatography (GC) analysis, as described by Jiménez Callejón et al. [68]. In brief, 10 mg of freeze-dried milled biomass were molturated with 10 mg of alumine for 5 min and stored at -21 °C until use. The molturated biomass was directly transesterified using 1 mL of acetyl chloride:methanol solution 1:20 vol/vol and 1 mL hexane. The reaction took place for 20 min at 105 °C and agitation. Afterwards, the mixture was left to reach room temperature, followed by the addition of 1 mL distil. water. The samples were then agitated and centrifuged. Two phases were formed, the upper one containing hexane and the produced FAMEs was removed and analyzed by GC. This was conducted in a Agilent Technologies 6890 N (Santa Clara, USA) GC, equipped with a capillary column of fused silica OmegaWax™ (0.25 mm x 30 m, 0.25 μm standard film, Supelco, Bellefonte, PA) and a flame ionization detector (FID). As carrier gas, nitrogen was used. Further technical details can be found in [68].

### Cascade processing of *S. almeriensis*

#### Carbohydrate analysis in water fraction

The water fraction containing the released carbohydrates was removed by centrifugation in a similar manner as described in 5.4.1. The samples were then stored in -20 °C until processing. After thawing, the carbohydrate content was determined following the same procedure with 5.2.3.

#### Enzymatic protein hydrolysis of *S. almeriensis*

Enzymatic hydrolysis was performed on wet biomass at concentration of 100 g·L^−1^, either directly after the PEF-treatment or, after centrifugation, removal of the supernatant and replacement by an equivalent volume of water (in case the water fraction was previously extracted, as in this case).

The protocol of enzymatic hydrolysis itself was described in full detail in [15]. In brief, enzymatic hydrolysis took place in 50 mL glass tubes with screw caps (Roth, Germany). Temperature was fixed at 50 °C in a water bath, placed atop a magnetic stirrer with heating function (neoLab, Germany) which also provided constant agitation. The pH was adjusted at 8 using sodium hydroxide (1 M). As enzymes, Alcalase (subtilisin) 2.5 L (Novozyme, Denmark) and Flavourzyme 1000 L (Novozyme, Denmark) were added at 3% (vol/w) each with regard to cell dry weight of the biomass. The hydrolysis reaction lasted for 3 h and samples were removed every 1 h with immediate deactivation of enzymes by heating at 80 °C for 10 min. Centrifugation at 10,000 × *g* for 10 min separated the water fraction along the free amino acids from the biomass and the amino acid content was determined spectrophotometrically using ortho-phtaldialdehyde (OPA) assay, with serine as standard. The ratio of the number of cleaved peptide bonds over the total number of peptide bonds in the sample, also defined as Degree of Hydrolysis (DH) offers an indication of the reaction rate. High pressure homogenization (HPH) was used as a benchmark. HPH took place in an EmulsiFlex-C3 homogenizer (Avestin Europe GmbH, Germany) at 2 MPa for 5 passes.

#### Lipid extraction from *S. almeriensis* following enzymatic hydrolysis

After 3 h of hydrolysis, approximately 3 mL microalgae suspension per sample for all conditions, were measured precisely into Teflon tubes. As mentioned in Sect. 5.5.2, the free amino acids were separated from the rest of the biomass through centrifugation. For lipid extraction, the following step of the cascade, the residual biomass pellet was re-suspended in 22.7 mL ethanol: hexane co-solvent 1: 0.41 vol/vol. Lipid extraction then took place as described in Sect. 5.4.1 (system A).

## Data Availability

All data generated or analyzed during this study are included in this published article.

## Abbreviations

PEF: Pulsed electric fields
HPH: High pressure homogenization
DW: Dry weight
